# Within-athlete association between morning on-track peak heart rate and same-day pre-performance salivary cortisol change under dry-track conditions: a multi-season single-case field study in a professional formula racing driver

**DOI:** 10.3389/fphys.2026.1813317

**Published:** 2026-08-24

**Authors:** Seiji Matsumura, Yuuki Ooishi, Makio Kashino, Naoki Saijo

**Affiliations:** Communication Science Laboratories, NTT, Inc., Atsugi, Japan

**Keywords:** endocrinology, heart rate, motorsport, physiological monitoring, salivary cortisol, single-case study, sports, wearable ECG

## Abstract

**Introduction:**

Professional Formula racing often requires multiple high-stakes on-track sessions within the same competition day, creating a need to characterize pre-performance physiological state under field constraints. Salivary cortisol is a widely used marker of hypothalamic–pituitary–adrenal axis activity, but field-ready options for its immediate assessment during competition remain limited. Wearable heart-rate monitoring during on-track driving may provide an immediately available field signal reflecting composite cardiovascular load. This multi-season single-case field study examined the within-athlete association between wearable heart-rate data and same-day pre-performance salivary cortisol change in one professional Formula racing driver.

**Materials and methods:**

The primary observational series comprised 23 race-day observations across three competitive seasons. During each morning on-track session, the driver wore a wearable electrocardiogram device, and the peak heart rate (HRpeak) was extracted. Saliva samples were collected approximately 30 and 10 min before each morning and afternoon session and assayed for cortisol. Within-day cortisol change (ΔCortisol) was defined as afternoon minus morning pre-session cortisol, averaged across the two samples. The primary analysis included 18 dry-track observations with complete HRpeak and cortisol data. Five additional observations were examined in an exploratory *post hoc* analysis.

**Results:**

In the primary analytical sample, morning HRpeak was positively associated with ΔCortisol (*r* = 0.720). The positive direction was retained in sensitivity checks using 5-s and 10-s average heart rates anchored on the HRpeak and addressing serial dependence, season/session characteristics, and −30 min and −10 min sample-specific cortisol changes. Including the single wet-track observation reduced the correlation to *r* = 0.316. In the *post hoc* analysis, standardized ΔCortisol residuals were negative for the three planned-timing nap opportunities. No inferential comparison was performed.

**Discussion:**

Wearable on-track HRpeak may provide an immediately available field signal of composite cardiovascular load associated with later same-day endocrine variation in the participating driver. This interpretation is limited to dry-track conditions and does not support the use of HRpeak as a stress-specific marker, prediction method, or decision rule; the association does not establish causality. The *post hoc* observations provide no evidence regarding nap efficacy. Nevertheless, these descriptive findings may inform future prospective studies.

## 1 Introduction

In elite sports, physical preparation alone is insufficient to achieve peak performance. Psychological readiness, particularly the ability to anticipate and manage stress, can influence competition outcomes (Hanton et al., 2009; Turner et al., 2020). Stress can impair performance not only through subjective discomfort, but also through disruptions in cognitive and motor control (Hardy, 1992; Lee et al., 2023). In precision-based disciplines such as motorsports, performance depends heavily on perceptual-cognitive expertise under dynamic, high-speed conditions, where attentional lapses may seriously compromise performance and safety (Lappi, 2018; Brown et al., 2019). Accordingly, field studies that characterize competition-day physiological variation are important for understanding athlete state under authentic performance constraints (Rumbold et al., 2012). In competition formats that require a demanding morning performance bout followed by other high-stakes bouts later on the same day, practitioners face a practical need to characterize later same-day pre-performance physiological state under field constraints. However, in applied settings, field evidence on competition-day physiological dynamics remains limited in some contexts, and existing knowledge does not always translate into timely, physiologically grounded, and field-ready methods under real competition schedules. This creates a need to evaluate associations between immediately available on-site measures and competition-day endocrine variation. Herein, we report a multi-season single-case field study designed to address this competition-day physiological assessment challenge in a real competitive setting.

Cortisol is one of the most well-validated and widely studied physiological indicators of hypothalamic–pituitary–adrenal (HPA) axis activity and stress-related endocrine regulation. It is secreted from the adrenal cortex via activation of the HPA axis and forms part of the endocrine stress response that supports metabolic and immune adaptation to challenge (Kirschbaum and Hellhammer, 1989; Ulrich-Lai and Herman, 2009). Although cortisol responses are adaptive in the acute context, prolonged stress-system activation may become maladaptive for health, recovery, and performance readiness (McEwen, 2007; Turner et al., 2020). Salivary cortisol is considered a reliable, noninvasive biomarker of HPA axis activity, with reproducible responses and well-documented temporal dynamics (Kirschbaum and Hellhammer, 1989). It offers a practical alternative to plasma assays, particularly in field-based sports sciences. However, interpreting salivary cortisol during a competition day is complicated by its strong diurnal rhythm and by multiple behavioral and field-contextual factors, including awakening time, nutrition, caffeine intake, sleep, travel, heat exposure, hydration status, physical activity, and other unmeasured competition-day influences (Pritchard et al., 2017; Tsunekawa et al., 2023). Accordingly, morning-to-afternoon cortisol change was treated in the present study as a same-day endocrine change, including possible deviation from the expected diurnal decline, rather than as a direct standalone measure of stress activation. In addition, field-ready options for immediate cortisol assessment during competition remain limited.

Heart-rate-derived measures may offer a practical approach to field monitoring. Heart rate variability (HRV) is commonly used for assessing autonomic cardiac regulation under standardized recording conditions. In athlete populations, HRV has also been examined alongside cortisol-related measures to characterize autonomic and endocrine stress regulation under observational or exercise-testing conditions (Kayacan et al., 2021, 2023). However, in real competition settings, practitioners also require simple, immediately interpretable signals that can be extracted during ongoing performance. The highest heart rate observed during an on-track session may provide one such field signal. Sport-related studies have used heart-rate-derived measures to characterize training load and autonomic cardiac regulation in athletes (Pichot et al., 2000), whereas heart rate has been measured alongside perceptual and metabolic variables during demanding sport-related tasks (Smith et al., 2015). During on-track Formula racing, however, peak heart rate should not be interpreted as a stress-specific marker. It may reflect multiple determinants, including physical and isometric workload, heat exposure, G-forces, braking and cornering behavior, respiratory pattern, driving intensity, vehicle dynamics, track condition, and psychological arousal (Schwaberger, 1987; Brown et al., 2019; Tornaghi et al., 2023; Tyler et al., 2026). Thus, morning on-track peak heart rate is best viewed as a pragmatic composite on-track load signal reflecting condition-dependent racing demands rather than psychological stress alone. Furthermore, heart rate data can also be acquired during competition using wearable technologies, supporting ecologically valid observation. Examining the within-athlete association between morning peak heart rate and same-day cortisol change may clarify whether this field measure covaries with later pre-performance endocrine variation. Although previous research has explored heart-rate-derived measures for training load, autonomic cardiac regulation, and stress-related adaptation in sport (Pichot et al., 2000; Turner et al., 2020), few studies have examined whether on-site peak heart-rate signals are associated with salivary cortisol change within the same competition day.

Within this conceptual framework linking wearable cardiovascular monitoring and same-day endocrine variation, professional Formula racing provides a suitable real-world setting for examining competition-day physiological monitoring. Formula racing drivers operate under high cognitive and physiological demand, and performance depends heavily on perceptual-cognitive expertise under high-speed, dynamic conditions (Schwaberger, 1987; Lappi, 2018; Brown et al., 2019; Tyler et al., 2026). Moreover, racing events often include multiple on-track sessions on the same day (e.g., a morning qualifying session and an afternoon final), providing natural opportunities for *in situ* physiological monitoring. These characteristics make professional Formula racing a valuable field context for studying within-athlete competition-day physiological patterns under authentic constraints.

The purpose of this study was to examine the within-athlete association between morning on-track peak heart rate and same-day pre-performance salivary cortisol change under dry-track conditions in one professional Formula racing driver. Given the single-case design, the study was intended to describe within-athlete race-day variation and inform future prospective studies rather than to provide population-level evidence, validate predictive utility, support a decision rule, or establish causality.

## 2 Materials and methods

### 2.1 Single-case design and reporting framework

This was a multi-season single-case observational field study. The primary observational series comprised 23 race-day observations across three competitive seasons and was used to examine the within-athlete association between morning on-track peak heart rate and same-day salivary cortisol change.

In light of single-case experimental design reporting principles, we note that the design did not include randomization, reversal, counterbalancing, alternating allocation, replication across participants, or a multiple-baseline structure (Tate et al., 2016). Therefore, the study should not be interpreted as a controlled single-case experimental design. A SCRIBE 2016-informed reporting checklist is provided in **Supplementary Table 1**.

### 2.2 Participant and ethics

This study involved one Japanese male elite professional Formula racing driver in his twenties. The participant had extensive competitive experience in the sport and was observed during official race-day sessions across three competitive seasons. The participant received no additional compensation for participating in the study.

To reduce re-identification risk, direct and nonessential indirect identifiers are not reported, including the participant’s exact age, team name, race-series name, country-specific series description, calendar years, round numbers, circuit names, detailed career history, and event-level descriptors that could link physiological observations to public competition records. Observations are indexed using anonymized event-order labels. These anonymization steps were intended to minimize, but cannot eliminate, residual re-identification risk.

As the study involved a human participant, it was reviewed and approved by the Ethics and Safety Committees of Communication Science Laboratories, NTT, Inc. (protocol number R03-002). The participant provided written informed consent prior to participation and was informed of the right to withdraw from the study at any time without penalty. The participant was informed that residual re-identification risk could not be completely eliminated and confirmed consent for publication of the reported physiological values and derived variables in de-identified form. All procedures were conducted in accordance with the principles outlined in the Declaration of Helsinki.

### 2.3 Physiological measures

#### 2.3.1 Salivary cortisol

Cortisol was measured in the participant’s saliva. Saliva samples (1 mL/collection) were collected via passive drool into 50-mL conical tubes. Sampling was performed twice per session, approximately 30 and 10 min before each session. These samples are referred to as the −30 min and −10 min pre-session samples. All samples were frozen immediately at −18 °C using a portable freezer at the racing venue. After each round, the portable freezer was transported to our laboratory, and the samples were transferred to an ultra-low-temperature freezer (−80 °C) until assay.

Salivary cortisol concentrations were quantified using liquid chromatography–tandem mass spectrometry. All analyses were conducted according to the standard protocols of ASKA Pharma Medical Co., Ltd. (Fujisawa, Japan) (Matsui et al., 2009; Yamashita et al., 2009), which are well established for various types of steroid hormone assays. Company personnel were blinded to both the sample contents and study details.

#### 2.3.2 Electrocardiograms

ECGs were used to measure the interbeat intervals (R–R intervals) throughout each on-track session. Analog data were amplified and digitized using a wearable ECG monitoring system (hitoe^®^, Toray Industries, Japan). The hitoe^®^ system is a textile-based biosensing platform that enables stable and low-noise ECG acquisition during physical activity (Yamaguchi and Togo, 2024) and has been used in sports measurement settings (Matsumura et al., 2021). The ECG data were transmitted in real time via Bluetooth to a smartphone mounted on the vehicle. The sampling rate was 200 Hz.

To calculate the R–R intervals from the ECG data, automatic R-wave detection and beat correction were performed using Kubios HRV Scientific software (version 4.2.0; Kubios Oy, Kuopio, Finland) with the software’s standard settings. The detected R waves and corrected R–R interval series were subsequently reviewed visually. Recordings were considered suitable for subsequent heart-rate analysis only when the morning-session ECG provided sufficient coverage and visual review showed no unresolved missed or extra R-wave detections in the segment used to derive the session-level peak heart-rate measure. No manual R-wave identification was required for any of the 18 observations included in the primary analysis.

### 2.4 Procedure

The primary observational series comprised 23 observations collected during official professional Formula racing events over three competitive seasons, including 22 dry-track observations and one wet-track observation. Calendar years, round numbers, circuit names, race-series identifiers, and team identifiers are not reported to reduce re-identification risk. Depending on the event, the schedule consisted of either a morning free-practice session followed by an afternoon qualifying session, or a morning qualifying session followed by an afternoon final race. Data from both race-day formats were analyzed. Morning session type and track condition were recorded from competition-day records and on-site field notes.

Saliva samples were collected approximately 30 and 10 min before each session. During the session, the driver wore the hitoe^®^ to record ECG data. After each session, the smartphone receiving ECG data from hitoe^®^ was removed from the vehicle, and the data were transferred to a laptop computer for analysis.

During four dry-track observations, the Bluetooth connection between the hitoe^®^ transmitter and the smartphone was interrupted, and automatic reconnection was unsuccessful, resulting in insufficient ECG data to derive the session-level peak heart-rate measure. The unusual high-speed racing environment, which included multiple concurrent wireless transmissions, may have contributed to the failure to reconnect; however, the cause could not be determined.

No specific controls were imposed on behavioral or lifestyle factors, such as caffeine consumption, nutrition, or sleep duration, to avoid disrupting the participant’s usual competition routines. However, these factors, as well as hydration status, temperature, session duration, driving intensity, travel, and other competition-context factors, were not systematically recorded or standardized, which is a limitation of the field design rather than a feature of the analysis.

### 2.5 Data analysis

For each morning on-track session, peak heart rate (HRpeak) was defined as the maximum of the five-beat moving-average heart-rate series computed by Kubios over the entire session, rather than as the athlete’s physiological maximal heart rate. The primary analytical sample comprised 18 dry-track observations with complete morning HRpeak and salivary cortisol data.

Cortisol concentrations from saliva samples collected approximately 30 and 10 min before starting each session were averaged to represent pre-session cortisol levels. Within-day cortisol change (ΔCortisol) was calculated as the difference between afternoon and morning cortisol (averaged afternoon cortisol minus averaged morning cortisol). Because cortisol typically declines from morning to afternoon, negative ΔCortisol values indicated a decline; values closer to zero indicated little or no decline, whereas positive values indicated an increase from morning to afternoon. ΔCortisol was interpreted as a same-day endocrine change, including possible deviation from the expected diurnal decline before the afternoon session. Because the −30 min and −10 min samples may contain information about short-term pre-session cortisol variation, the two samples were also reported separately, and within-session cortisol slopes were calculated descriptively as the −10 min value minus the −30 min value divided by 20 min. The averaged pre-session value was used as a session-level summary of two closely spaced field samples, not as evidence that the two sampling times were physiologically interchangeable. A Bland–Altman-style mean–difference plot was used to describe morning-to-afternoon pre-session cortisol differences, with 95% limits of agreement calculated as the mean difference ± 1.96 SD of the differences. The Pearson correlation between the paired mean and the afternoon-minus-morning difference was also calculated.

Primary statistical analyses were performed using JASP (version 0.95.4; JASP Team, The Netherlands). In the primary analytical sample, the within-athlete association between morning HRpeak and ΔCortisol was summarized using the Pearson correlation coefficient and an unadjusted ordinary least-squares slope. The fitted relationship should not be interpreted as an independently validated prediction model or decision rule. As supporting analyses, Pearson correlations were calculated between morning HRpeak and the averaged morning and afternoon pre-session cortisol concentrations and between morning HRpeak and the separate −30 min and −10 min morning cortisol concentrations. The primary association was visualized using a scatter plot with the fitted unadjusted ordinary least-squares regression line. Because all observations were repeatedly obtained from a single athlete, correlation coefficients, regression coefficients, residuals, and diagnostic statistics were interpreted descriptively as within-athlete summaries rather than as population-level inferential estimates.

For the HRpeak averaging-window sensitivity analyses, fixed 5-s and 10-s windows were centered on the third beat of the five-beat HRpeak block. Mean heart rate within each window was calculated by dividing 60 by the mean of the valid, artifact-corrected beat-level R–R intervals within that window after expressing the intervals in seconds. The Pearson correlation coefficient and unadjusted ordinary least-squares slope were recalculated using these HRpeak-centered 5-s and 10-s mean heart rates. In a separate sensitivity analysis of cortisol sampling time, the Pearson correlation coefficient and unadjusted ordinary least-squares slope for the HRpeak–ΔCortisol association were recalculated using ΔCortisol values derived separately from the −30 min and −10 min samples.

Additional sensitivity analyses addressing the repeated-observation structure and serial dependence were performed using Python (version 3.13.5) with pandas, NumPy, statsmodels, and Matplotlib. Lag-1 autocorrelations were calculated for morning HRpeak and ΔCortisol. Residual serial dependence from the unadjusted model was assessed using the Durbin–Watson statistic and a Ljung–Box test at lag 1. A generalized least-squares model with a first-order autoregressive error structure was fitted using observation order as the temporal index. Descriptive sensitivity models were fitted with adjustment for season code alone and for season code plus morning session type.

To address ECG recording failures, the four dry-track observations with unavailable morning HRpeak were summarized descriptively using their available salivary cortisol data. Their salivary cortisol values were also compared descriptively with the range of values observed in the primary analytical sample. These observations could not contribute to HRpeak-based analyses, and missing HRpeak values were not imputed because doing so would require unverifiable assumptions about unobserved on-track heart-rate responses.

The single wet-track observation occurred during free practice and was not pooled into the primary analysis because the physiological meaning of HRpeak may differ by track condition. Specifically, wet-track conditions may differ from dry-track conditions in speed profile, grip, braking and cornering behavior, vehicle dynamics, physical and cardiovascular demands, attack-lap behavior, and risk-management strategy. In a track-condition sensitivity analysis, the Pearson correlation was recalculated after adding the wet-track observation to the primary sample.

## 3 Results

### 3.1 Analytical sample and observation structure

The primary observational series comprised 23 race-day observations across three competitive seasons. The corresponding dataset included anonymized event-order labels, season code, morning session type, track condition, salivary cortisol values, and morning on-track HRpeak.

Of the 23 observations, 22 were conducted under dry-track conditions and one under wet-track conditions. Morning on-track HRpeak was unavailable for four dry-track observations because of interruptions in Bluetooth transmission, leaving 18 dry-track observations with complete HRpeak and salivary cortisol data for the primary analysis. The single wet-track observation occurred during free practice and was analyzed separately. The observation flow and analytical samples are summarized in **Table 1**.

**Table 1:** Observation flow and analytical samples.

Observation category	Track condition	Total observations	Season/event labels included	Morning session type, n	Morning HRpeak available	Salivary cortisol available	Analytical handling
Primary observational series	Dry/Wet	23	S1: E1–E10; S2: E1–E9; S3: E1–E4	FP: 13; QS: 10	19	23	Source series for the primary analysis
Primary analytical sample	Dry	18	S1: E1–E5, E8–E10; S2: E2–E4, E6, E8–E9; S3: E1–E4	FP: 10; QS: 8	18	18	Used for the primary HRpeak–ΔCortisol association
Dry-track observations with unavailable HRpeak	Dry	4	S1: E7; S2: E1, E5, E7	FP: 2; QS: 2	0	4	Reported separately using cortisol data only; no HRpeak imputation
Wet-track observation	Wet	1	S1: E6	FP: 1	1	1	Reported separately because HRpeak interpretation was not assumed to be equivalent to dry-track observations and included only in the track-condition sensitivity analysis

The table summarizes the 23 race-day observations in the primary observational series. The primary analytical sample comprised 18 dry-track observations with complete morning on-track HRpeak and salivary cortisol data. Four dry-track observations with unavailable morning on-track HRpeak because of Bluetooth transmission interruptions and the single wet-track observation were reported separately and were not pooled into the primary HRpeak-based analysis. S1–S3 indicate anonymized season codes, and E indicates event order within each season. FP indicates free practice, and QS indicates qualifying session.

### 3.2 Salivary cortisol profile and morning-to-afternoon cortisol change

In the primary analytical sample of 18 dry-track observations, morning pre-session cortisol averaged across the −30 min and −10 min samples was 2.69 ± 0.82 ng/mL, and afternoon pre-session cortisol was 2.93 ± 1.38 ng/mL. The mean afternoon-minus-morning cortisol change was 0.24 ± 1.15 ng/mL. Twelve of the 18 observations showed a positive morning-to-afternoon change, whereas six showed a negative change.

When the −30 min and −10 min samples were examined separately, morning cortisol was 2.79 ± 0.90 ng/mL at −30 min and 2.60 ± 1.00 ng/mL at −10 min. Afternoon cortisol was 2.79 ± 1.56 ng/mL at −30 min and 3.08 ± 1.34 ng/mL at −10 min. Accordingly, the afternoon-minus-morning change was 0.00 ± 1.50 ng/mL for the −30 min samples and 0.48 ± 1.18 ng/mL for the −10 min samples. The within-session slope was −0.0095 ± 0.0482 ng/mL/min before morning sessions and 0.0143 ± 0.0461 ng/mL/min before afternoon sessions. These values show the components of the averaged pre-session cortisol measure and the short-term variation between the two sampling times.

**Figure 1:**
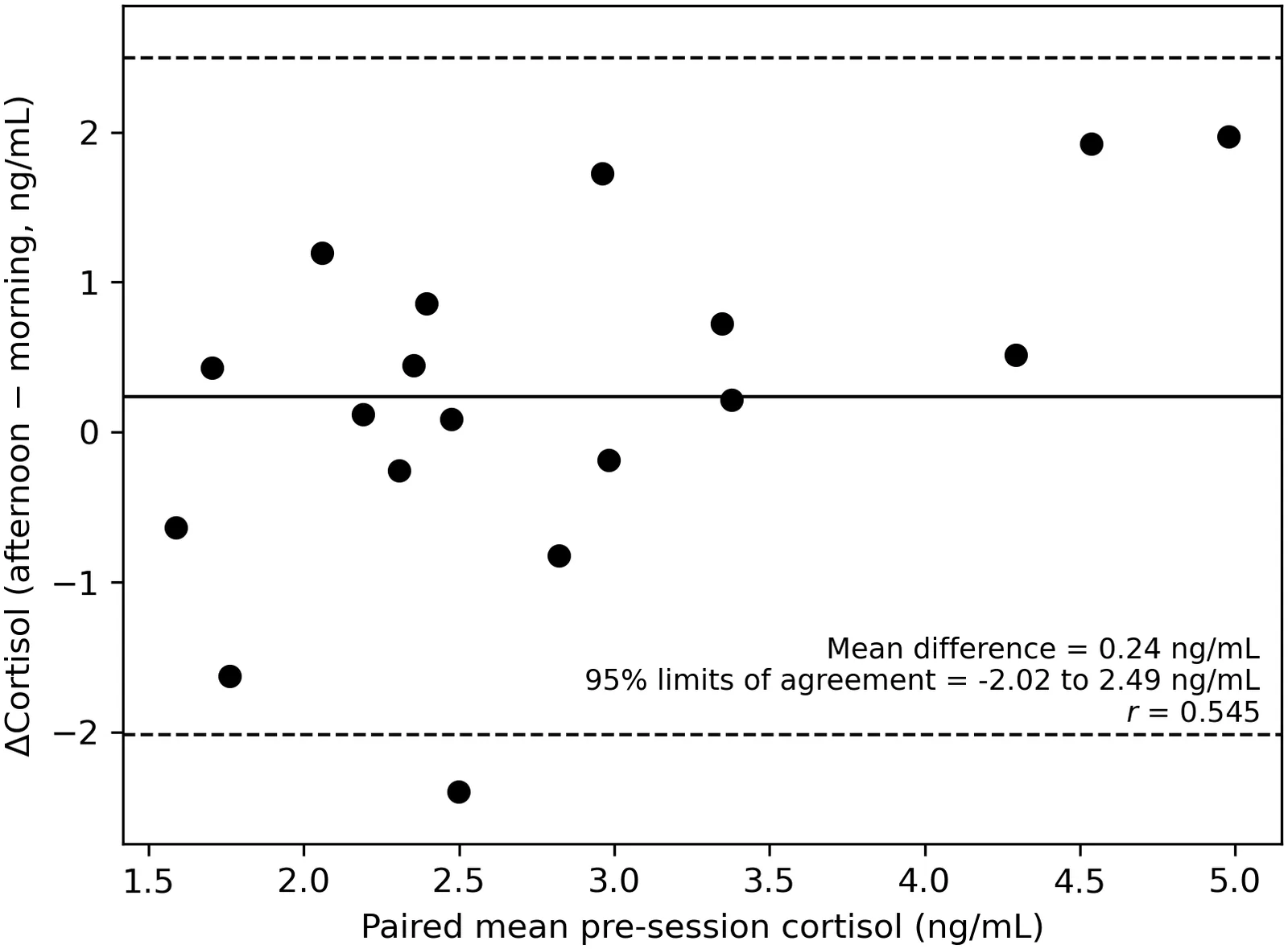
Bland–Altman-style comparison of morning and afternoon pre-session salivary cortisol values in the primary analytical sample. Each point represents one of the 18 dry-track race-day observations with complete morning on-track HRpeak and salivary cortisol data. The x-axis shows the paired mean of morning and afternoon pre-session salivary cortisol concentrations, each averaged across the −30 min and −10 min samples. The y-axis shows the afternoon-minus-morning pre-session cortisol difference, defined as ΔCortisol based on these averaged values. The solid horizontal line indicates the mean difference, and the dashed horizontal lines indicate the 95% limits of agreement. Positive values indicate higher afternoon than morning pre-session cortisol, whereas negative values indicate the reverse. The *r* value indicates the correlation between the paired mean and the afternoon-minus-morning difference and is reported as a descriptive measure of the relationship between overall pre-session cortisol level and morning-to-afternoon cortisol change.

A Bland–Altman-style comparison of morning and afternoon pre-session cortisol values showed a mean afternoon-minus-morning difference of 0.24 ng/mL, with 95% limits of agreement from −2.02 to 2.49 ng/mL (**Figure 1**). The paired mean was positively associated with the afternoon-minus-morning difference (*r* = 0.545), indicating that larger morning-to-afternoon cortisol differences tended to occur on days with higher overall pre-session cortisol levels. This descriptive pattern does not identify whether the difference was driven primarily by morning values, afternoon values, or other unmeasured factors. Summary values for the averaged cortisol measures, separate −30 min and −10 min samples, and within-session slopes are shown in **Table 2**.

**Table 2:** Salivary cortisol profile in the primary analytical sample.

Measure	Morning	Afternoon	ΔCortisol (afternoon – morning)
−30 min sample cortisol (ng/mL)	2.79 ± 0.90	2.79 ± 1.56	0.00 ± 1.50
−10 min sample cortisol (ng/mL)	2.60 ± 1.00	3.08 ± 1.34	0.48 ± 1.18
Averaged pre-session cortisol (ng/mL)	2.69 ± 0.82	2.93 ± 1.38	0.24 ± 1.15
Within-session slope (ng/mL/min)	−0.0095 ± 0.0482	0.0143 ± 0.0461	—

Values are presented for the 18 dry-track observations with complete morning on-track HRpeak and salivary cortisol data. Morning and afternoon pre-session cortisol values are shown separately for the −30 min and −10 min samples and as their average. ΔCortisol was calculated as afternoon pre-session cortisol minus morning pre-session cortisol for each sampling definition. Within-session cortisol slopes were calculated as the −10 min value minus the −30 min value divided by 20 min and are reported separately for morning and afternoon sessions. The afternoon-minus-morning difference was not calculated for within-session slopes. Values are reported as mean ± SD. Cortisol concentrations are expressed in ng/mL, and slopes in ng/mL/min.

These descriptive results indicate that the −30 min and −10 min samples should not be assumed to represent the same pre-session cortisol state. Accordingly, the averaged pre-session cortisol value was used only as a session-level summary, and the HRpeak–ΔCortisol association was also checked using ΔCortisol values calculated separately from the two samples.

### 3.3 Primary dry-track association between morning HRpeak and same-day cortisol change

**Figure 2:**
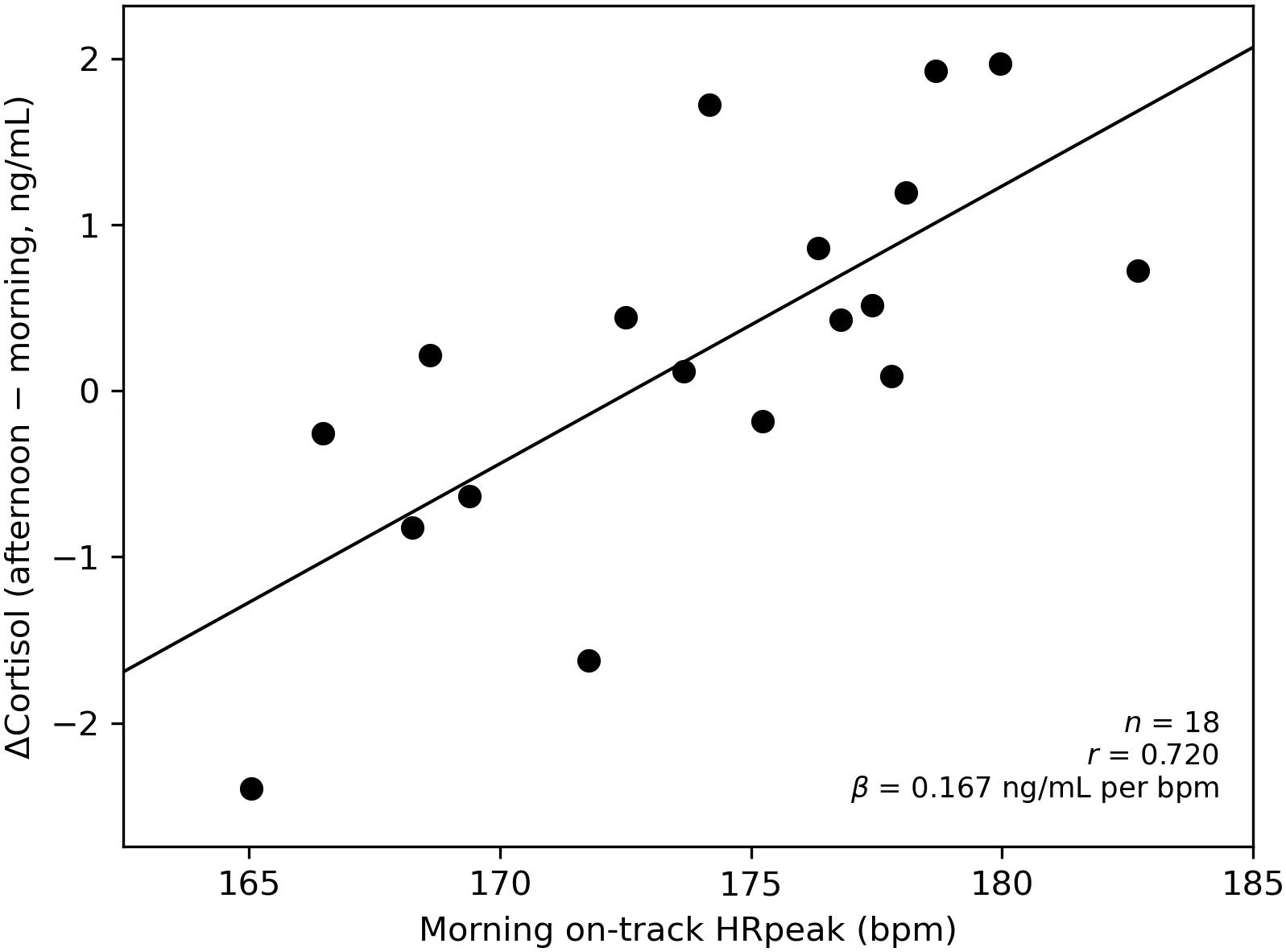
Association between morning on-track HRpeak and same-day salivary cortisol change in the primary analytical sample. Each point represents one of the 18 dry-track race-day observations with complete morning on-track HRpeak and salivary cortisol data. ΔCortisol was calculated as afternoon pre-session cortisol minus morning pre-session cortisol, with each pre-session value averaged across the −30 min and −10 min samples. Higher ΔCortisol values indicate greater deviation from the expected morning-to-afternoon cortisol decline. The fitted line represents the unadjusted ordinary least-squares regression in the primary analytical sample, and β indicates the corresponding unadjusted slope.

In the primary analytical sample, morning on-track HRpeak showed a positive within-athlete association with same-day ΔCortisol (**Figure 2**). The descriptive Pearson correlation coefficient was *r* = 0.720. In the corresponding linear model, the unadjusted slope was 0.167 ng/mL per bpm.

As supporting context, morning on-track HRpeak showed essentially no association with morning pre-session cortisol (*r* = −0.001), whereas it was positively associated with afternoon pre-session cortisol (*r* = 0.602). This pattern suggests that the HRpeak association was more evident for later same-day pre-session cortisol than for the morning value itself. The corresponding descriptive correlations were also examined separately for the −30 min and −10 min morning cortisol samples (−30 min: *r* = 0.090; −10 min: *r* = −0.083), indicating that the absence of a clear HRpeak–morning cortisol association was not dependent solely on the averaged morning cortisol definition.

As a sampling-time-specific sensitivity analysis, the HRpeak–ΔCortisol association was also examined using the −30 min and −10 min samples separately. The positive association was retained for both the −30 min and −10 min sample-based ΔCortisol. For the −30 min sample-based ΔCortisol, the descriptive Pearson coefficient was *r* = 0.661, and the unadjusted slope was 0.201 ng/mL per bpm. For the −10 min sample-based ΔCortisol, the descriptive Pearson coefficient was *r* = 0.563, and the unadjusted slope was 0.134 ng/mL per bpm.

### 3.4 Sensitivity analyses and observations not included in the primary analysis

Sensitivity analyses addressing the HRpeak averaging window, repeated observations, and season/session characteristics are summarized in **Table 3**. The positive association with ΔCortisol was retained when 5-s and 10-s average heart rates anchored on the five-beat HRpeak were used (5 s: *r* = 0.735, unadjusted slope = 0.181 ng/mL per bpm; 10 s: *r* = 0.715, unadjusted slope = 0.166 ng/mL per bpm). The lag-1 autocorrelation was −0.196 for morning on-track HRpeak and −0.132 for ΔCortisol. Residual serial-dependence diagnostics from the unadjusted model showed no clear positive temporal autocorrelation; the Durbin–Watson statistic was 2.37, and the Ljung–Box test at lag 1 was not significant. The Durbin–Watson statistic and lag-1 autocorrelations were more consistent with a mild negative than a positive first-order structure, although this pattern does not by itself invalidate the descriptive estimates from the unadjusted ordinary least-squares model.

**Table 3:** Sensitivity analyses addressing the HRpeak averaging window, repeated observations, and season/session characteristics.

Analysis	Model/diagnostic	Estimate/statistic	Interpretation
HRpeak-anchored 5-s average heart rate	Pearson correlation and unadjusted OLS slope	r = 0.735; slope = 0.181 ng/mL per bpm	Positive association retained
HRpeak-anchored 10-s average heart rate	Pearson correlation and unadjusted OLS slope	r = 0.715; slope = 0.166 ng/mL per bpm	Positive association retained
Morning HRpeak	Lag-1 autocorrelation	−0.196	No clear positive adjacent-observation autocorrelation
ΔCortisol	Lag-1 autocorrelation	−0.132	No clear positive adjacent-observation autocorrelation
Unadjusted model residuals	Durbin–Watson statistic	2.37	No clear positive residual serial dependence
Unadjusted model residuals	Ljung–Box test at lag 1	*Q* = 1.101; *p* = 0.294	No clear lag-1 residual autocorrelation
Averaged ΔCortisol	AR(1)-GLS sensitivity model	*β* = 0.176 ng/mL per bpm	Positive HRpeak coefficient retained
Averaged ΔCortisol	Season-adjusted descriptive model	*β* = 0.164 ng/mL per bpm	Positive HRpeak coefficient retained
Averaged ΔCortisol	Season + morning session type-adjusted descriptive model	*β* = 0.162 ng/mL per bpm	Positive HRpeak coefficient retained

The table summarizes sensitivity analyses using HRpeak-anchored 5-s and 10-s average heart rates, and diagnostic and sensitivity analyses addressing the repeated-observation structure of the single-case dataset. Lag-1 autocorrelations were calculated for morning on-track HRpeak and ΔCortisol. Residual serial dependence from the unadjusted model was assessed using the Durbin–Watson statistic and the Ljung–Box test at lag 1. The HRpeak–ΔCortisol association was further examined using an AR(1)-GLS model with observation order as the temporal index and descriptive models adjusted for season code and morning session type. For the Ljung–Box test, *Q* was evaluated against a *χ²* distribution with 1 degree of freedom at lag 1.

In an AR(1)-GLS sensitivity model using observation order as the temporal index, the positive HRpeak–ΔCortisol association was retained, with an HRpeak coefficient of 0.176 ng/mL per bpm. A season-adjusted model yielded a positive HRpeak coefficient of 0.164 ng/mL per bpm. A model adjusted for both season and morning session type likewise retained a positive HRpeak coefficient. These sensitivity checks do not eliminate all concerns about non-independence in this single-case dataset, but they provide no clear evidence that the primary HRpeak–ΔCortisol association was driven solely by adjacent-observation serial dependence, season, or morning session type. Because the adjusted models included season code and morning session type in a sample of only 18 observations, they should be regarded as exploratory and constrained by the number of available observations.

The four dry-track observations with unavailable morning HRpeak had complete salivary cortisol data and were summarized descriptively to document observations not included in HRpeak-based analyses. Their cortisol values overlapped with the range observed in the primary analytical sample, although the missing-HRpeak observations were concentrated within a narrower and generally lower part of the afternoon cortisol and ΔCortisol ranges. Using the same averaged pre-session cortisol definition as in the primary analysis, the missing-HRpeak observations showed ranges of 1.45–2.47 ng/mL for morning cortisol, 1.22–2.28 ng/mL for afternoon cortisol, and −0.97 to 0.83 ng/mL for ΔCortisol; the corresponding ranges in the primary analytical sample were 1.46–4.04 ng/mL, 0.95–5.97 ng/mL, and −2.40 to 1.97 ng/mL. Thus, these observations were not excluded because of outlying cortisol values, but because morning HRpeak was unavailable. However, given the lower and narrower afternoon cortisol and ΔCortisol ranges in these observations, non-random missingness related to competition-day physiological state cannot be ruled out. Including them in the HRpeak–ΔCortisol analysis would have required imputing unobserved HRpeak values, which was not performed because such imputation would require unverifiable assumptions about on-track heart-rate responses in this single-athlete field dataset.

The single wet-track observation occurred during a wet-track free-practice session and had a morning HRpeak of 124.02 bpm, which was well below the dry-track HRpeak range of 165.05–182.71 bpm. It was summarized separately for the reasons described in the Methods. In the track-condition sensitivity analysis, adding the wet-track observation to the dry-track sample substantially attenuated the descriptive Pearson correlation from *r* = 0.720 to *r* = 0.316.

### 3.5 Exploratory *post hoc* analysis

Short daytime naps may support alertness and aspects of cognitive or motor performance and represent a field-feasible option between same-day competition sessions (Waterhouse et al., 2007; Faraut et al., 2015). In an exploratory *post hoc* analysis, five additional dry-track race-day observations, separate from the 23-observation primary series, were examined after a brief between-session nap opportunity had been proposed.

The intended nap opportunity was scheduled approximately 30 min before the afternoon session, with an intended duration of approximately 10–15 min. When applicable, the nap opportunity was provided inside a trailer in a quiet environment with minimal disturbances. Use of the nap opportunity and its timing were recorded from the competition-day schedule and on-site confirmation; however, sleep onset and sleep duration were not objectively verified using polysomnography or actigraphy. Based on these records, the five observations comprised three planned-timing nap observations, one early-timing nap observation, and one no-nap observation. These observations were not randomized or counterbalanced. Therefore, they were not treated as intervention groups, and no effect size or inferential comparison was calculated. ECG and salivary cortisol data were processed as described above. Manual R-wave identification was required for one of the five observations.

For descriptive context only, the unadjusted ordinary least-squares regression fitted to the 18 primary dry-track observations served as a within-athlete reference to calculate standardized residuals for the five additional observations. For each observation, the residual was calculated as the observed ΔCortisol minus the value fitted from morning HRpeak and was standardized by dividing it by the residual standard error of the reference model. Negative and positive standardized residuals indicated observations below and above the fitted reference line, respectively. This reference relationship was not an independently validated prediction model.

Standardized residuals were negative for all three planned-timing nap observations (−2.89, −0.81, and −3.43), positive for the early-timing nap observation (0.98), and close to zero for the no-nap observation (0.08). Although all three planned-timing nap observations had negative standardized residuals, one observation was only modestly below the primary dry-track reference line, whereas the other two were more negative.

These residuals describe how the additional observations deviated from the primary dry-track HRpeak–ΔCortisol reference relationship. They were not used to estimate nap efficacy, compare intervention groups, or validate a decision rule. Accordingly, they should be regarded as descriptive field observations rather than as evidence that brief naps altered cortisol dynamics.

## 4 Discussion

### 4.1 Summary of findings

This multi-season single-case field study used a pragmatic competition-day approach to examine the within-athlete association between morning on-track HRpeak and same-day pre-performance salivary cortisol change in one professional Formula racing driver. The main finding was that morning on-track HRpeak was positively associated with same-day morning-to-afternoon salivary cortisol change under dry-track conditions. Because salivary cortisol typically declines from morning to afternoon (Weitzman et al., 1971; Adam et al., 2017), positive or near-zero ΔCortisol values were interpreted as deviations from the expected diurnal decrease under competition conditions. Thus, higher morning on-track HRpeak was associated with greater same-day deviation from this expected decline.

This finding represents a within-athlete observational pattern rather than population-level evidence. Sensitivity checks addressing the HRpeak averaging window, adjacent-observation serial dependence, AR(1) error structure, season, and morning session type retained the positive direction of the association. These checks reduce, but do not eliminate, concerns arising from repeated observations within a single athlete. In contrast, adding the wet-track observation substantially attenuated the correlation, indicating that the observed association was sensitive to the inclusion of the wet-track observation and, therefore, to the analytical sample.

An exploratory *post hoc* analysis of additional dry-track observations was descriptive only and did not provide evidence of nap efficacy.

### 4.2 Interpretation of competition-day heart-rate and cortisol signals

Morning on-track HRpeak should be interpreted as a composite on-track load signal rather than as a stress-specific marker. During professional Formula racing, peak heart rate may reflect physical and isometric workload, heat exposure, G-forces, braking and cornering behavior, respiratory pattern, driving intensity, vehicle dynamics, track condition, and psychological arousal (Schwaberger, 1987; Brown et al., 2019; Tyler et al., 2026). The observed HRpeak–ΔCortisol association therefore indicates a within-athlete relationship between a composite cardiovascular load signal during the morning session and later same-day endocrine variation; it does not identify a specific autonomic–endocrine mechanism.

This interpretation is consistent with the finding that morning HRpeak showed essentially no association with morning pre-session cortisol, whereas it was positively associated with afternoon pre-session cortisol and ΔCortisol. This pattern is consistent with HRpeak reflecting on-track determinants that were not captured by pre-session cortisol alone. Accordingly, the data are best interpreted as an empirical within-athlete association between a composite competition-day cardiovascular signal and later same-day cortisol variation, not as evidence of a stress-specific causal pathway.

The present data do not establish an operational HRpeak threshold, prediction method, decision rule, or competition-day action algorithm. The observed HRpeak–ΔCortisol association should be regarded as a descriptive within-athlete relationship, not as a validated prediction model. Any future use of morning HRpeak to inform between-session decisions would require prospective validation, prespecified outcomes, objective evaluation of false-positive and false-negative decisions, and replication in additional athletes or controlled single-case designs.

Previous field studies in high-demand performance contexts have measured heart rate and cortisol under authentic competitive or naturalistic conditions, including professional motorcycling and extreme-sport settings such as skydiving (Filaire et al., 2007; Hare et al., 2013; Meyer et al., 2015). These studies underscore the broader value of collecting cardiovascular and endocrine measures in real-world performance environments. Determining whether the observed association generalizes beyond the present athlete will require prospective studies with additional athletes, standardized contextual covariate collection, repeated observations across dry- and wet-track conditions, and analytical designs capable of separating cardiovascular load, environmental demands, and psychological arousal.

### 4.3 Interpretation of salivary cortisol change and track-condition specificity

Salivary cortisol follows a strong diurnal rhythm, with generally higher levels in the morning and a decline across the day; therefore, morning-to-afternoon changes must be interpreted relative to the expected diurnal decrease (Weitzman et al., 1971; Adam et al., 2017). In the present dataset, many race days showed positive or near-zero ΔCortisol, indicating that afternoon pre-session cortisol often deviated from the expected morning-to-afternoon decrease. ΔCortisol should not be interpreted as a direct stress-specific response because several behavioral and field-contextual factors were not systematically recorded, including awakening time, breakfast timing, caffeine intake, prior-night sleep, heat exposure, travel logistics, and physical workload (Pritchard et al., 2017; Ushiki et al., 2020).

The Bland–Altman-style plot suggested that larger ΔCortisol values tended to occur on days with higher overall pre-session cortisol levels. This descriptive mean–difference pattern may partly reflect shared dependence on overall pre-session cortisol level. Because the field-contextual factors noted above were not systematically recorded, this descriptive pattern does not identify the specific source of higher afternoon pre-session cortisol.

Track condition may also affect the physiological interpretation of on-track HRpeak. As noted in the Methods, wet-track free-practice sessions may differ from dry-track sessions in speed profile, grip, braking and cornering behavior, vehicle dynamics, physical and cardiovascular demands, attack-lap behavior, and risk-management strategy. Because the wet-track observation occurred during free practice, the contributions of track condition, session type, and session-specific driving strategy to the lower HRpeak could not be disentangled. In the present dataset, the wet-track observation had a morning HRpeak well below the dry-track HRpeak range and was summarized separately because the physiological meaning of HRpeak was not assumed to be equivalent across track conditions. Including this observation in the track-condition sensitivity analysis substantially attenuated the correlation. Future studies should prospectively stratify dry- and wet-track sessions rather than assuming equivalence across track conditions.

Overall, ΔCortisol is best understood in this study as a same-day pre-performance endocrine change under competition conditions, including deviation from the expected morning-to-afternoon decline. The HRpeak–ΔCortisol association suggests a within-athlete relationship between morning on-track cardiovascular load and later same-day endocrine variation, but it does not establish a stress-specific mechanism.

### 4.4 Exploratory *post hoc* analysis of a brief between-session nap opportunity

The exploratory *post hoc* analysis provided descriptive observations related to a brief between-session nap opportunity. The standardized residuals were negative for all three planned-timing nap observations, positive for the early-timing nap observation, and close to zero for the no-nap observation. This pattern may help inform future prospective studies, but it should not be interpreted as evidence of nap efficacy.

The *post hoc* analysis included only five observations, nap timing was not randomized or counterbalanced, and sleep onset and sleep duration were not objectively verified. Concurrent influences such as vehicle development, driver adaptation, weather, track characteristics, championship context, accumulated fatigue, and unmeasured behavioral factors could not be separated from nap timing. Therefore, the residuals should be interpreted only as descriptive field observations relative to the primary dry-track reference relationship. Future studies should test brief between-session nap opportunities using prospective designs with objective sleep monitoring and adequate control observations.

### 4.5 Limitations and future directions

Given the single-case design, these findings cannot be generalized across broader athletic populations. Accordingly, the present findings should be interpreted as descriptive within-athlete observations rather than as population-level evidence. Additional studies with more athletes are needed because the magnitude of the association between HRpeak and within-day cortisol change may differ across athletes depending on autonomic reactivity, HPA-axis responsiveness, fitness level, heat tolerance, sleep status, caffeine use, driving style, and competition context.

From a single-case experimental design perspective, the present study did not establish experimental control (Tate et al., 2016). The study lacked randomization, reversal, counterbalancing, alternating allocation, replication across participants, and a multiple-baseline structure. Therefore, the exploratory *post hoc* analysis cannot establish a functional relation between the proposed brief between-session nap opportunity and cortisol dynamics.

The statistical approach also has limitations. Pearson correlation and residual analyses were used to describe the within-athlete HRpeak–ΔCortisol relationship across race days, but they do not fully account for potential temporal dependence among repeated observations from the same athlete. Although lag-1 autocorrelation, residual serial-dependence diagnostics, an AR(1)-GLS sensitivity model, and season/session-adjusted models were included, the small number of observations limits model stability. Future studies should use prospectively specified longitudinal, time-series, or controlled single-case designs with denser and more regularly spaced observations.

Although the cortisol values of the four dry-track observations with unavailable morning HRpeak overlapped with the range observed in the primary analytical sample, their afternoon cortisol and ΔCortisol values were generally lower and less variable. Non-random missingness therefore cannot be excluded, and the absence of these observations may have affected the magnitude of the observed association. The primary HRpeak–ΔCortisol association remains conditional on the availability of valid morning ECG-derived HRpeak data. Track-condition inference was likewise limited because only one wet-track observation was available, precluding characterization of differences between dry- and wet-track conditions.

Behavioral, environmental, and competition-specific covariates were not systematically recorded or controlled. This is a major limitation, because these contextual factors can influence heart rate or cortisol. Salivary cortisol measurements are influenced by biological and lifestyle factors, including daily rhythm and caffeine ingestion (Pritchard et al., 2017). Therefore, the observed association between morning HRpeak and within-day cortisol change cannot be attributed specifically to psychological stress or anticipatory arousal. Ecological validity does not remove the need for covariate collection; the absence of systematic covariate recording is a design limitation. Future prospective studies should preserve the authentic competition setting while systematically recording key contextual factors, including awakening time, breakfast timing, caffeine dose and timing, prior-night sleep duration and quality, hydration and nutritional intake, ambient and cockpit temperature, humidity, session duration, driving intensity, track condition, circuit-specific technical demands, lap count, race pace, attack-lap status, telemetry-derived workload indicators, travel logistics, championship context, vehicle settings, driving strategy, and race-day incidents.

Another limitation is that HRV-derived indices were not included in the primary analysis, despite HRV being a widely used marker of autonomic cardiac regulation under standardized recording conditions. Previous athlete studies have examined HRV alongside cortisol-related measures, suggesting that HRV may provide complementary information when recordings are obtained under appropriate conditions (Kayacan et al., 2021, 2023). However, the ECG recordings in the present study were obtained during high-intensity, non-stationary on-track driving rather than during standardized resting or steady-state conditions. Rapid changes in speed, braking, cornering, G-forces, respiratory pattern, and upper-body muscle activity may limit the interpretability of HRV-derived indices during racing. In addition, HRV metrics vary with exercise intensity and time, which may further reduce their interpretability during rapidly changing exercise conditions (Brockmann and Hunt, 2023). Future studies would benefit from collecting standardized resting or pre-session ECG segments, which would allow time-domain, frequency-domain, or nonlinear HRV indices to be examined alongside HRpeak.

The cortisol sampling design also limits the interpretation of HPA-axis dynamics. Although ΔCortisol was used to partially account for day-level baseline differences and the expected morning-to-afternoon decline, it cannot fully separate the circadian decrease, anticipatory activation, physical workload, and unmeasured behavioral influences. In addition, because the present analysis focused on pre-session cortisol samples, the full time course of HPA-axis activity across the competition day could not be evaluated. Standardized diurnal salivary cortisol assessment generally requires repeated sampling at key diurnal time points across multiple days and attention to protocol adherence (Hulett et al., 2019), and exercise-stress assessment using salivary cortisol may require sequential sampling within the circadian rhythm (Ushiki et al., 2020). Future studies should include more frequent sampling across the day, including post-session measurements, to better characterize the temporal dynamics of cortisol responses. The present analysis separated the −30 min and −10 min samples and reported within-session slopes, but these two pre-session samples still provide only a limited view of cortisol dynamics.

Despite these limitations, the present multi-season single-case field study provides detailed within-athlete observations from an ecologically valid elite professional Formula racing setting. Future research should test whether the dry-track HRpeak–cortisol-change association replicates within and across athletes, prospectively stratify dry- and wet-track sessions, record contextual and behavioral covariates, include standardized ECG segments suitable for HRV analysis, and evaluate brief between-session nap opportunities using objective sleep monitoring and prespecified allocation procedures.

### 4.6 Conclusion

In conclusion, this multi-season single-case field study found a positive within-athlete association between morning on-track HRpeak and same-day pre-performance salivary cortisol change under dry-track conditions in one professional Formula racing driver. The positive direction was retained across sensitivity checks, whereas including the wet-track observation substantially attenuated the correlation. HRpeak should be regarded as a composite on-track load signal rather than as a stress-specific marker. The observed association should not be interpreted as a validated prediction model or decision rule and does not establish causality. These findings represent descriptive single-athlete observations that may inform future prospective studies.

## Data Availability

The datasets presented in this article are not readily available because they cannot be deposited in a public repository due to ethical and institutional data security restrictions. Researchers who wish to access the data for academic purposes can contact the corresponding author upon reasonable request. Requests to access the datasets should be directed to Seiji Matsumura, seiji.matsumura@ntt.com.
