# Supplementary material for: Within-athlete association between morning on-track peak heart rate and same-day pre-performance salivary cortisol change under dry-track conditions: a multi-season single-case field study in a professional formula racing driver
Source: Front Physiol. 2026 Aug 24;17:1813317. doi: 10.3389/fphys.2026.1813317 (PMC13547096; doi:10.3389/fphys.2026.1813317)
Supplement: Supplementary file 1 [file Table1.pdf]

# *Supplementary Material*

**Supplementary Table 1.** SCRIBE 2016-informed reporting checklist for the multi-season single-case observational field study. The checklist is used as a reporting transparency framework and should not be interpreted as a claim that the study met criteria for a controlled single-case experimental design (SCED). Items requiring planned intervention phases, randomization, reversal, counterbalancing, or replication are reported as not performed or as design limitations.

| Item | SCRIBE area | Status | Where reported | Study-specific report for supplement |
|---|---|---|---|---|
| 1 | Title | Reported | Title page; Abstract | The title and abstract identify the work as a multi-season single-case field study of the within-athlete association between morning on-track HRpeak and same-day pre-performance salivary cortisol change under dry-track conditions. The study is not described as a controlled SCED. |
| 2 | Abstract | Reported | Abstract | The abstract reports the single-athlete design, the 23-observation primary series, the 18-observation dry-track analytical sample, the principal association, sensitivity analyses, the single wet-track result, and the five additional observations examined post hoc. |
| 3 | Scientific background | Reported | Introduction | The introduction explains the rationale for examining whether an immediately available on-track heart-rate signal covaries with same-day pre-performance cortisol change in elite motorsport and identifies HRpeak as a composite on-track load signal rather than a stress-specific marker. |
| 4 | Aims | Reported | Introduction, final paragraph; Results: Exploratory post hoc analysis | The primary aim is to examine the within-athlete association between morning on-track HRpeak and same-day pre-performance salivary cortisol change under dry-track conditions. The nap-related observations are introduced separately as an exploratory post hoc analysis and are not part of the primary aim. |
| 5 | Design | Reported with limitation | Methods: Single-case design and reporting framework; Discussion: Limitations and future directions | The primary series is a multi-season single-case observational field study, not a phase-based experiment. It includes no randomization, reversal, counterbalancing, alternating allocation, multiple-baseline structure, or replication across participants. Five later observations were examined separately only in a post hoc analysis. |
| 6 | Procedural changes | Reported with clarification | Results: Exploratory post hoc analysis | No procedural change formed part of the 23-observation primary series. A brief between-session nap opportunity proposed during five later observations is described only in the exploratory post hoc analysis. |
| 7 | Replication | Not performed | Methods: Single-case design and reporting framework; Discussion: Limitations and future directions | Repeated observations were obtained from one athlete across three competitive seasons, but there was no replication across participants or experimental phase sequences. |

| Item | SCRIBE area | Status | Where reported | Study-specific report for supplement |
|---|---|---|---|---|
| **8** | Randomization | Not performed | Methods: Single-case design and reporting framework; Results: Exploratory post hoc analysis | No randomization or counterbalancing was used. The five post hoc observations were not treated as randomized intervention groups. |
| **9** | Blinding | Partially reported | Methods: Salivary Cortisol; Results: Exploratory post hoc analysis | Assay company personnel were blinded to the sample contents and study details. The participant and field staff could not be blinded to the nap opportunity; no controlled intervention comparison was made. |
| **10** | Selection criteria | Partially reported | Methods: Participant and ethics | The study involved one elite professional Formula racing driver observed by the research team. No population sampling frame or recruitment process for a SCED series was used. |
| **11** | Participant characteristics | Reported | Methods: Participant and ethics | Sex, nationality, approximate age category, elite professional status, and competitive experience are reported. More granular characteristics and event identifiers are withheld to reduce re-identification risk. |
| **12** | Setting | Reported | Methods: Participant and ethics; Procedure | Data were collected during official professional Formula racing events across three competitive seasons. Event and circuit identifiers are withheld to reduce re-identification risk; morning session type and track condition were recorded. |
| **13** | Ethics | Reported | Methods: Participant and ethics; Data availability statement | The manuscript reports institutional approval, written informed consent, the right to withdraw, anonymization of nonessential identifiers, residual re-identification risk, and consent for publication of physiological values and derived variables in de-identified form. |
| **14** | Measures | Reported | Methods: Physiological Measures; Data analysis | The manuscript reports salivary cortisol sampling and LC–MS/MS assay procedures; ECG acquisition; Kubios R-wave detection, beat correction, and visual review; the five-beat HRpeak definition; HRpeak-anchored 5-s and 10-s sensitivity measures; and the frequency of manual R-wave identification. |
| **15** | Equipment and materials | Reported | Methods: Physiological Measures; Procedure; Data analysis | The manuscript identifies the hitoe® wearable ECG system, Bluetooth-connected smartphone, saliva collection and storage equipment, LC–MS/MS assay provider, Kubios HRV Scientific, JASP, and Python-based analysis software. |
| **16** | Intervention or phase exposure | Reported with limitation | Results: Exploratory post hoc analysis; Discussion: Exploratory post hoc analysis | Five additional observations are described post hoc after a brief between-session nap opportunity had been proposed. Intended timing and duration are reported, but sleep onset and duration were not objectively verified. These observations were not treated as a controlled intervention phase. |
| **17** | Procedural fidelity | Limited | Results: Exploratory post hoc analysis; Discussion: Exploratory post hoc analysis | Use and timing of the nap opportunity were documented from the competition-day schedule and on-site confirmation. No actigraphy, polysomnography, or formal fidelity measure was used. |

| Item | SCRIBE area | Status | Where reported | Study-specific report for supplement |
|---|---|---|---|---|
| **18** | Analytic methods | Reported | Methods: Data analysis; Results | The manuscript reports the primary dry-track Pearson correlation and unadjusted linear slope; HRpeak-anchored 5-s and 10-s sensitivity analyses; sampling-time-specific cortisol analyses; serial-dependence diagnostics; AR(1)-GLS and season/session models; wet-track and missing-data handling; and descriptive post hoc residuals. |
| **19** | Sequence completed | Reported | Results: Analytical sample and observation structure; Table 1; Exploratory post hoc analysis | The primary series comprised 23 observations: 22 dry-track and one wet-track. Four dry-track observations lacked HRpeak, leaving 18 observations for the primary analysis. Five additional dry-track observations were examined separately in the post hoc analysis. |
| **20** | Outcomes and estimation | Reported with restriction | Results; Figures; Data availability statement | The results report the primary within-athlete association, averaging-window and repeated-observation sensitivity analyses, the wet-track sensitivity result, missing-observation summaries, and descriptive post hoc residuals. The relationship is not presented as a validated prediction model, decision rule, or causal effect. Event-level data are restricted because of re-identification risk. |
| **21** | Adverse events | Not systematically assessed | Not specifically reported in the manuscript | Adverse-event monitoring was not a prespecified study outcome, and no systematic adverse-event analysis was performed. |
| **22** | Interpretation | Reported | Discussion | The discussion interprets the findings as descriptive within-athlete observations under dry-track conditions. HRpeak is treated as a composite on-track load signal rather than a stress-specific marker, and no prediction, decision, causal, or nap-efficacy claim is made. |
| **23** | Limitations | Reported | Discussion: Limitations and future directions | Reported limitations include one participant, repeated observations, four missing HRpeak records and possible non-random missingness, one wet-track observation, unrecorded contextual factors, limited cortisol timing, and lack of randomization and objective sleep verification for the post hoc observations. |
| **24** | Applicability | Reported | Discussion: Interpretation, limitations, and future directions; Conclusion | Applicability is limited to descriptive within-athlete observations in the monitored driver and related field settings. No population-level generalization, validated prediction method, or decision rule is asserted. |
| **25** | Protocol | Not preregistered | Methods: Single-case design and reporting framework; Supplementary Table 1 | No public prospectively registered protocol specified a controlled SCED or the post hoc nap analysis. The absence of randomization, reversal, and replication is reported, and additional analytic checks are described as sensitivity analyses. |
| **26** | Funding | Reported | Funding statement | The manuscript reports support from JST Moonshot R&D (Grant Number JPMJMS2295) and states that the funders had no role in study design, data collection and analysis, publication decision, or manuscript preparation. |
